# Normal Variants, Pitfalls, and Artifacts in Ga-68 Prostate Specific Membrane Antigen (PSMA) PET/CT Imaging

**DOI:** 10.3389/fnume.2022.825512

**Published:** 2022-02-21

**Authors:** Nico Malan, Mboyo-di-Tamba Vangu

**Affiliations:** Division of Nuclear Medicine and Molecular Imaging, Department of Radiation Sciences, University of the Witwatersrand, Johannesburg, South Africa

**Keywords:** prostate cancer, positron emission tomography/computer tomography, Ga68-prostate specific membrane antigen, PSMA, variants, pitfalls, normal biodistribution, PET/CT

## Abstract

The advent of gallium 68 prostate specific membrane antigen (PSMA) PET imaging has revolutionized the diagnosis and treatment of prostate cancer. PSMA is a transmembrane glycoprotein that is overexpressed in prostate cancer and yields images with high tumor-to-background contrast. Effective “one-stop-shop” imaging of the prostate, lymph nodes, soft tissue, and bone is achieved with PSMA PET. Compared to conventional imaging, PSMA PET provides superior sensitivity and specificity and plays a pivotal role in staging high-risk prostate cancer as well as in biochemical recurrence by identifying oligometastatic disease. PSMA PET furthermore assists in the selection of patients with metastatic castrate resistant prostate cancer for possible treatment (e.g., labeled with a beta emitter lutetium 177) by using a theranostic approach. The term “prostate specific” is a misnomer as PSMA is also present in other malignant and benign conditions since it acts as a folate hydrolase. To avoid pitfalls and false-positives, a sound knowledge of the normal biodistribution of PSMA as well as other potential causes for false-positive uptake is imperative. This review will describe the expected patterns of distribution of Ga 68 PSMA PET imaging and the common pitfalls noted in published literature since the topic is still evolving.

## Introduction

The advent of PET/CT (positron emission tomography/computer tomography) has revolutionized medical imaging. Not only does it provide for anatomical detail but merged onto the anatomical images is a molecular map of the specific disease process being interrogated. F18-FDG (18F-fluoro-2-deoxy-D-glucose) has been the main work horse of PET imaging and was originally synthesized at Brookhaven National Laboratory in Upton, New York in the United States in 1976.

F18-FDG PET/CT has a low reported sensitivity for prostate cancer (PCa) due to the low avidity of F18-FDG in most prostate cancer cells (1). In contrast, the advent of Ga-68 PSMA PET/CT has revolutionized the diagnosis and treatment of prostate carcinoma. Prostate-specific membrane antigen is a type II transmembrane glycoprotein and is overexpressed in prostate cancer cells. PSMA is encoded by the folate hydrolase 1 (FOLH1) gene. FOLH1 is also referred to as the glutamate carboxypeptidase II (GCPII) gene (2).

Ga68-PSMA-11 was first used by the Heidelberg Group in in-human case series and is the most common used agent today. The synthetization of Ga68-PSMA-11 is achieved through labeling the precursor PSMA-11 by using the chelator N,N-bis(2-hydroxybenzyl) ethylenediamine-N,N diacetic acid with Ga68 (2).

To increase production and imaging capacity, F18 PSMA-1007 is now available (3). However, when we mention PSMA in this paper, we allude to Ga 68-PSMA-11 since it has been widely used and accepted as reference measure of PSMA overexpression in individuals with prostate carcinoma.

In contrast to other specific tracers (like iodine) where higher grade tumors show decreased sensitivity due to dedifferentiation of the tumor, PSMA expression is increased in dedifferentiated/high grade tumors (4).

Therapeutic applications are possible given the endocytosis mediated internalization following PSMA ligand binding. It is specifically the apical membrane rather than the cytoplasmic membrane PSMA expression that is significantly enhanced in prostate cancer cells (4). There is currently no specific threshold PSMA ligand uptake [standardized uptake value (SUV)] to reliably differentiate between PCa and other cells (4).

It is important to understand that, although contrary to the name “prostate-specific,” PSMA expression is not specific to the prostate cells, but is also found in other cells. The normal physiological biodistribution of PSMA resembles the distribution of PSMA tissue expression as well as non-specific excretion of PSMA.

It is worth noting that other PET tracers exist to image prostate cancer (F18-NaF, C11-acetate, C11/F18 Choline, amino acid analogs (F18-FACBC), F18 dihydrotestosterone analogs) (5).

PSMA has however outperformed standard imaging and has the potential to evaluate response to therapy based on theranostics (6). A sound knowledge of the normal biodistribution of PSMA is essential for correct image interpretation and reporting.

## Normal Biodistribution of PSMA

**Figure 1:**
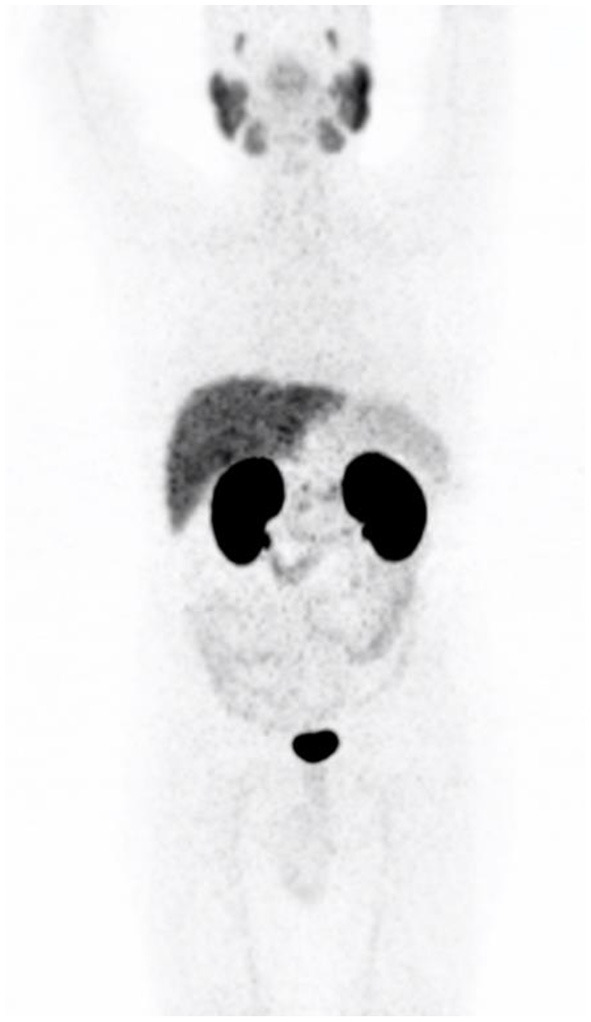
Maximal intensity projection image, showing the normal biodistribution of Ga68-PSMA.

Normal physiological PSMA uptake is demonstrated in the lacrimal glands, parotid, and submandibular salivary glands, liver, spleen, bowel (especially small bowel and specifically the duodenum), as well as renal activity with subsequent renal clearance through the ureters, urinary bladder, and urethra (see Figure 1). As with other nuclear medicine radiotracers, PSMA is also excreted through saliva and this explains possible esophageal, stomach, or laryngeal activity. Ga68-PSMA also undergoes hepatobiliary clearance, and thus, activity may be appreciated in the bile ducts, gall bladder, and extra-hepatic ducts.

The most intense activity is appreciated in the kidneys with subsequent urinary excretion (ureters and urinary bladder) as well as lacrimal and salivary glands. The high-grade uptake in small bowel, particularly the duodenum, is likely attributed to the dietary uptake of folates in this region and thus increased PSMA expression. Relatively low-grade activity is appreciated in the liver and spleen, and this activity should be uniform in distribution (2, 4, 6).

Low grade physiological activity is also appreciated in sympathetic ganglia (celiac, stellate, hypogastric, and presacral ganglia). This is more commonly visualized with newer PETCT scanners with the incorporation of time-of-flight. Knowledge of the location of sympathetic ganglia is important as these may be misinterpreted as possible metastatic nodal disease. The stellate ganglia are typically located para-vertebral at the level of the thyroid, the coeliac ganglia are seen para-aortic at the level of the kidneys, the hypogastric ganglia are located para-vertebral at the level of the iliac wings, and the sacral ganglia are in the pre-sacral area. Physiological activity may also be seen in the trigeminal ganglia which is located within the trigeminal (Meckel) cave. The pancreas may demonstrate heterogenous and variable uptake in relation to the variable number, distribution, and volume of islet cells which have been reported to express PSMA receptors (2, 4, 6–8).

**Figure 2:**
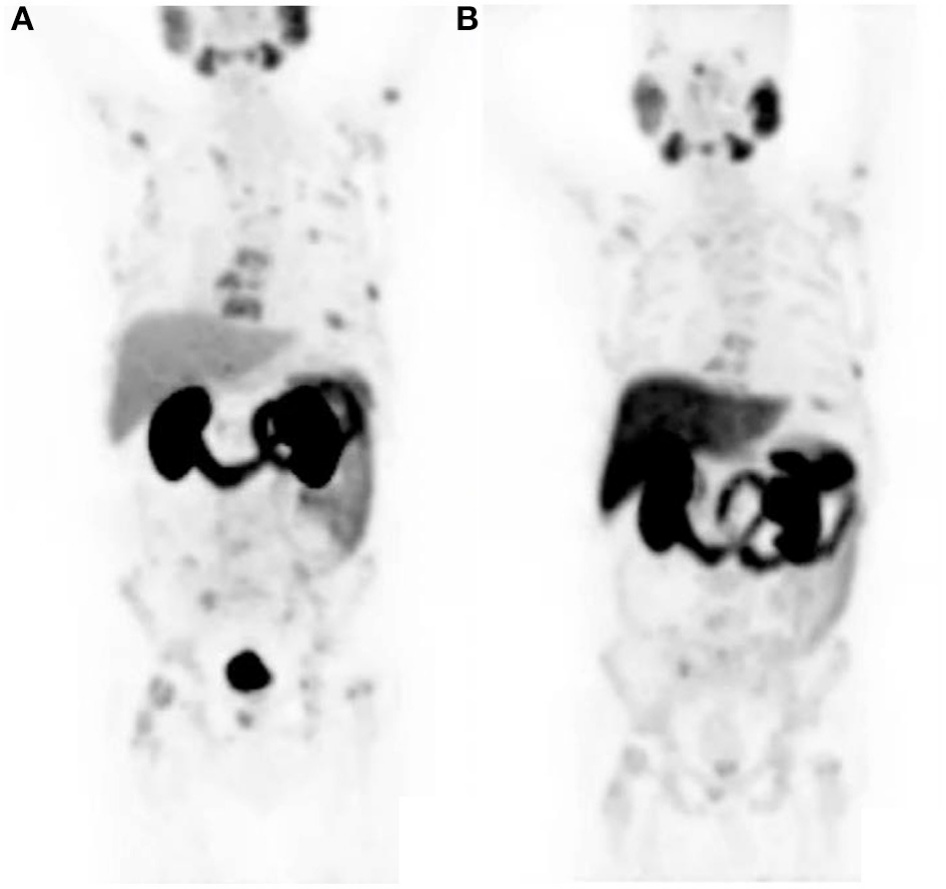
The same patient received both Ga68-PSMA-11 and F18-PSMA-1007 a day apart. Notice the lesser hepatobiliary activity and greater urinary activity on the Ga68-PSMA-11 study **(A)** and greater hepatobiliary activity and lesser urinary activity on the F18-PSMA-1007 study **(B)**. Both studies show a similar pattern of metastatic bone lesions.

As alluded to earlier, both F11-PSMA-1007 and Ga68-PSMA-11 are available, and it is worth noting that the normal biodistribution between the two radiopharmaceuticals differ. Ga68-PSMA-11 shows greater urinary excretion and lesser hepatobiliary activity as opposed to F18-PSMA-1007 which shows greater hepatobiliary activity and lesser urinary excretion (see Figure 2).

## Distribution of Metastatic Sites in Patients With Prostate Cancer

**Figure 3:**
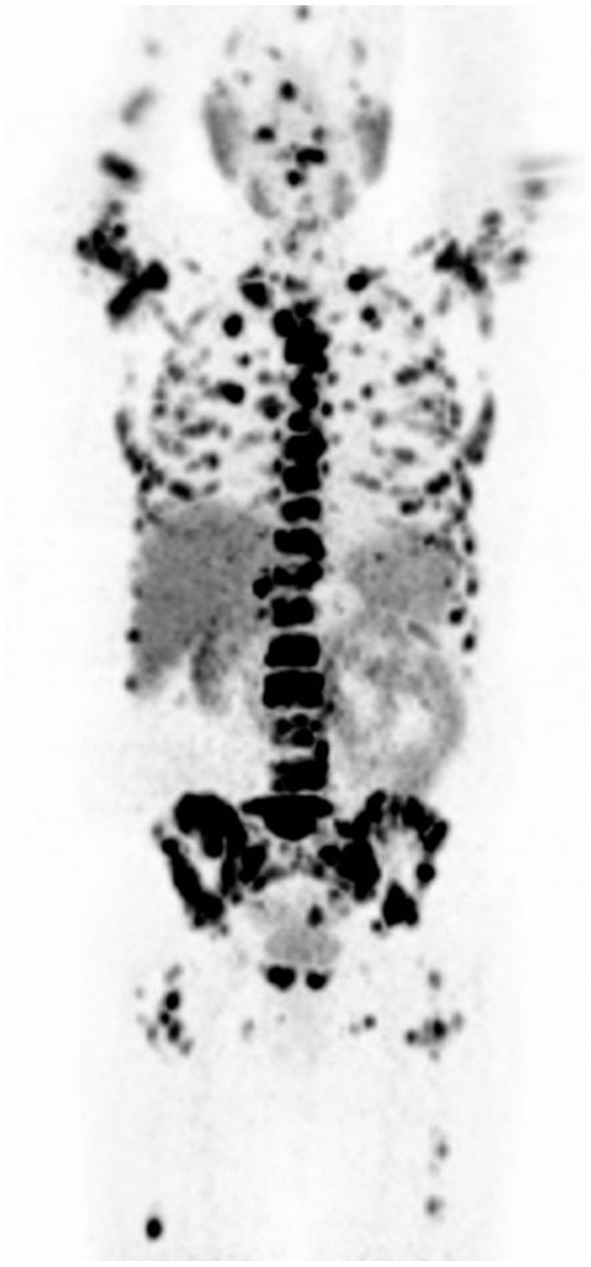
Maximal intensity projection image showing widespread osteoblastic metastases in a castrate resistant prostate carcinoma patient.

Gandaglia et al. (9) described the distribution of the most common metastatic sites of prostate cancer in a population of 74,826 patients. The most common site was bone (84%) (see Figure 3) followed by distant lymph nodes (10.6%) and liver (10.2%). It was noted that there were also atypical sites identified—brain, the digestive system, kidneys, lungs, and adrenal glands.

It must be emphasized that careful assessment of the supra-clavicular area is essential as nodal spread in this area is common even in the absence of nodal metastases in the upper abdomen. This is thought to be attributed to the drainage pathway of the left thoracic duct (2).

Vlachostergios et al. (10) also highlights that PSMA expression is associated with prostate carcinoma grade and stage and has a relationship with androgen receptor signaling. They assigned an imaging score to PSMA uptake and showed that patients with a higher imaging score (i.e., higher PSMA uptake) had a significantly shorter overall survival of 15.8 months (95% CI: 13.0–18.1 months) compared to those with low PSMA expression and thus lower PSMA uptake (22.7 months (95% CI: 17.7–30.7 months). They concluded that PSMA uptake is an independent prognostic factor for overall survival. 68Ga PSMA uptake was also shown to correlate with plasma PSA levels as well as an association with more aggressive biological behavior of the tumor.

PSMA imaging plays a pivotal role in locally advanced prostate cancer and in cases of biochemical recurrence since PSMA is upregulated in >90–100% of prostate cancer cells. It therefore is classified as “strong level of evidence” by the European Association of Urology to utilize PSMA PET-CT in any case of biochemical recurrence (PSA > 0.2 ng/ml) after radical prostatectomy, since this receptor based radiopharmaceutical results in superior sensitivity and high inter-reader agreement as compared to choline and fluciclovine. High risk patients which are hormone-sensitive and with biochemical recurrence with oligo-metastatic disease present a problematic population since other imaging procedures may not detect disease and PSMA is ideally suited in this population. PSMA imaging also has good performance and is also able to detect approximately two-thirds of disease sites in patients with biochemical persistence (PSA > 0.1 ng/ml, 6 weeks post-surgery) (11).

## PSMA Uptake in Non-prostate Cancer

### Infection/Inflammation

There is currently little known regarding immune cell PSMA expression. Possible postulated mechanisms include neovascularization, macrophage folate receptors, and increased vascular flow and permeability (4).

Non-prostatic inflammatory or infectious processes are usually easily appreciated when assessing the corresponding morphological CT changes. This may include neurocysticercosis, tuberculosis, diverticulosis, and post-surgical inflammatory changes and others.

Furthermore, inflammatory PSMA uptake may also be seen in the prostatic bed and prostatic urethra following radical prostatectomy or radiation therapy and should not be interpreted as disease recurrence. This is usually appreciated within 2 months of the treatment, and assessment of morphological features including surrounding fatty stranding is essential and findings should be correlated with the clinical history of the patient (2, 4).

Specificity of PSMA prostatic uptake is degraded by moderate PSMA uptake in benign conditions such as prostatitis, granulomatous disease, and benign prostatic hyperplasia. This is thought to be as a result of using the background prostate activity as threshold when deciding if lesions are PET-positive lesions in some studies (12). Caution should thus be exercised when assessing the prostate gland when there is no formal histological diagnosis of prostate cancer.

**Figure 4:**
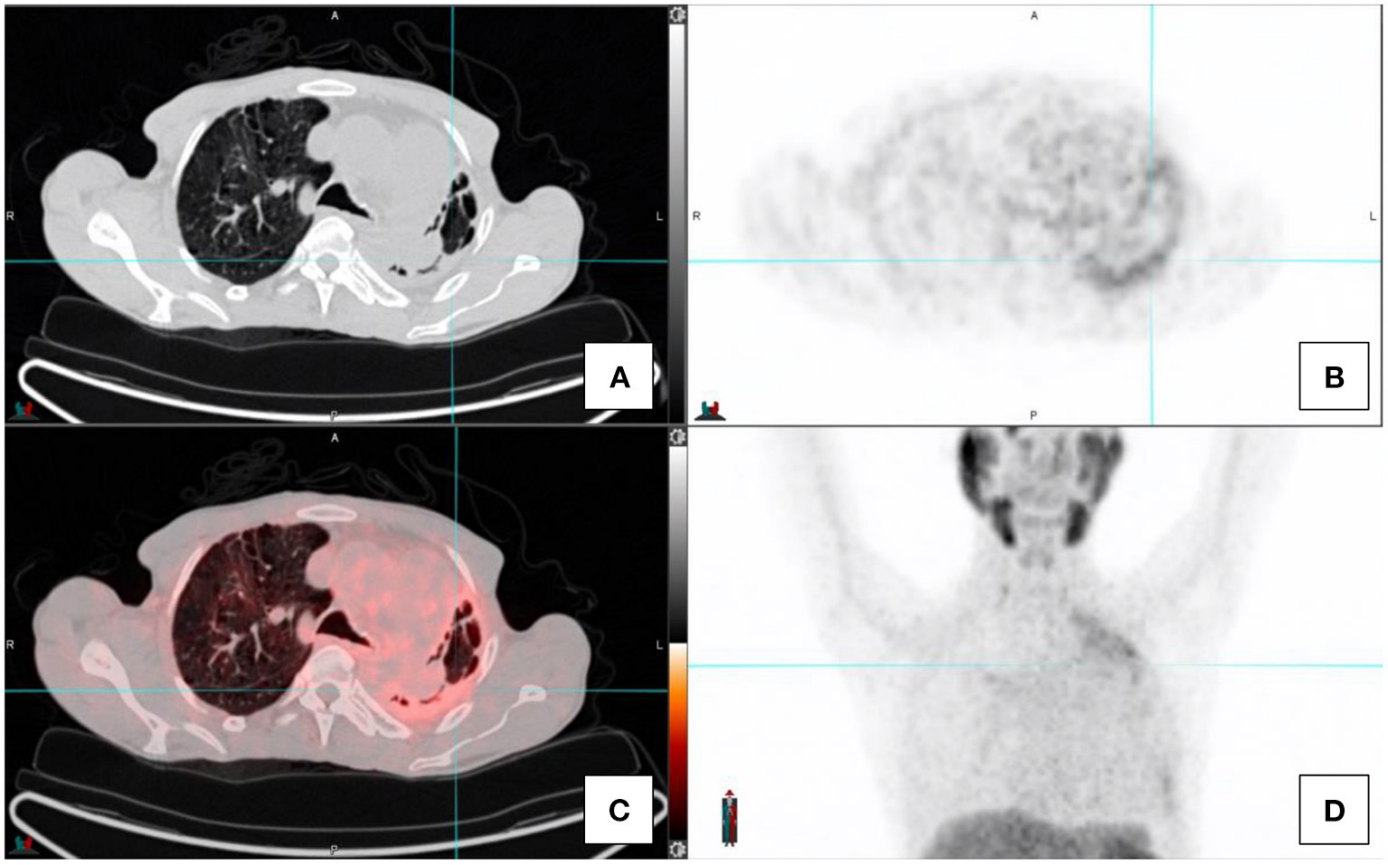
Transaxial images in a prostate cancer patient with concomitant fibro-cavitatory pulmonary tuberculosis. The patient was recently started on treatment for tuberculosis. CT-image **(A)**, PET image **(B)**, fused PET/CT image **(C)**, and the maximal intensity projection image **(D)**.

Inflammatory and infective pulmonary entities are frequently encountered in the lungs (see Figure 4). These usually demonstrate low grade PSMA activity and are morphologically distinct to the multiple solid pulmonary nodules of varying size and higher PSMA uptake that characterize pulmonary metastases or the nodular pleural thickening of the pleura which indicates pleural involvement from prostate cancer. Single ground-glass surrounded nodules with low grade PSMA uptake are usually associated with inflammatory or infections processes (2, 4, 13).

### Bone Conditions

Bone metastases are the most common site of metastatic disease. This usually presents as sclerotic foci on CT component with associated increased PSMA uptake. In aggressive prostate cancer, lytic bone metastatic lesions may be encountered with associated increased PSMA uptake (2, 4, 13).

It is important to assess the morphological features on CT component as non-benign bone conditions may also exhibit PSMA uptake. Generally, the associated uptake in metastatic prostate cancer bone lesions is higher than that of benign pathology. Osteophytic endplate changes is commonly seen in patients undergoing Ga68 PSMA scan for prostate cancer. This is usually easily appreciated when assessing the morphological changes, i.e., disc narrowing and end plate osteophytic changes. A single rib PSMA focus, in the absence of morphological changes, similar to bone scan requires clinical correlation as this may be attributed to trauma. Bone marrow metastases may occur, and these may exhibit focal high grade uptake without associated CT changes (2, 4, 13).

**Figure 5:**
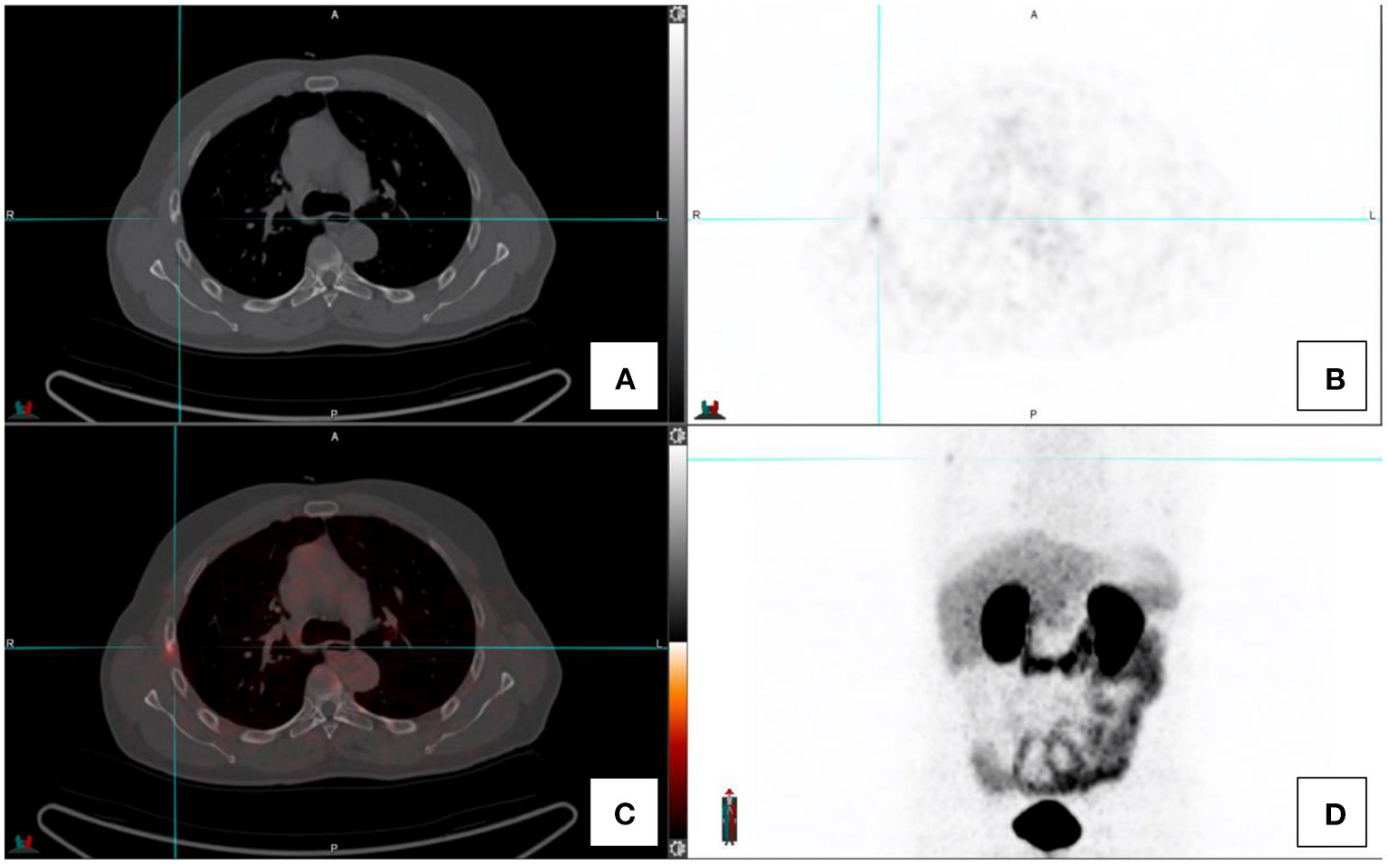
Transaxial images in a prostate cancer patient with a right sided rib fracture. CT-image **(A)**, PET image **(B)**, fused PET/CT image **(C)**, and the maximal intensity projection image **(D)**.

A variety of bone related conditions have been reported to present with PSMA uptake and include osteomyelitis, fractures, Paget's disease, fibrous dysplasia, hemangioma, and others (see Figure 5). As the PSMA uptake in hemangiomas may be variable, assessment of morphological features is essential. Hemangiomas typically present with thickened trabeculae or vascular lacunae but sometimes may present with atypical morphological features (2, 4, 13).

### Benign Neoplasms

There has been increasing reports of PSMA uptake in benign neoplasms. This was noted to involve soft tissues and abnormal vascular proliferation. PSMA uptake was noted in some instances of meningiomas, nerve sheath tumors, and other neurogenic lesions. Soft tissue lesions included thyroid and parathyroid adenomas, thymomas, adrenal adenomas, dermatofibromas, and others. These lesions are usually easily differentiated as being unrelated to prostate cancer in view of the specific location or morphological characteristics adrenal adenomas usually present as hypodense lesions in the adrenal glands (Hounsfield unit <10) (2, 4, 13).

**Figure 6:**
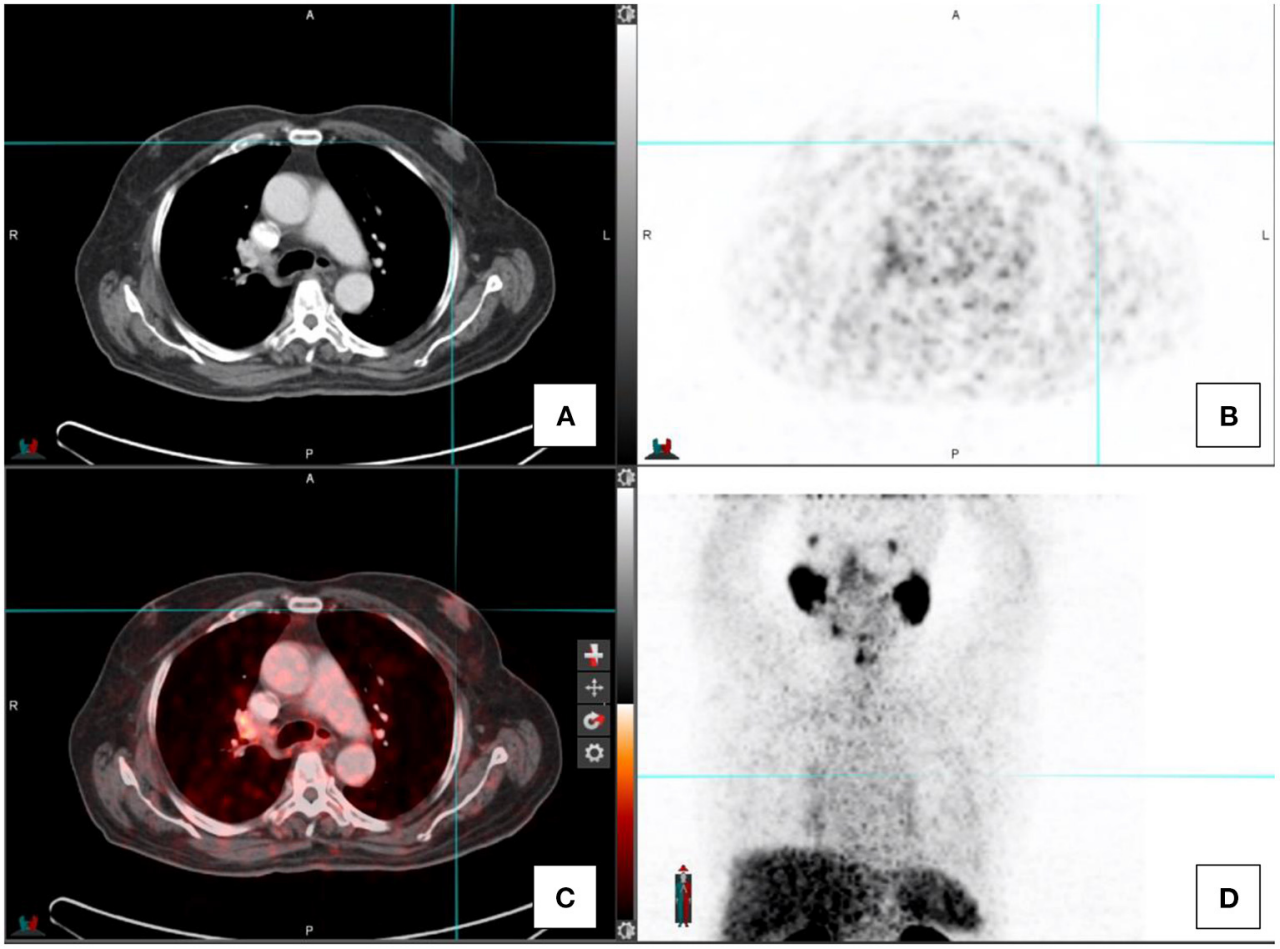
A patient with gynecomastia with associated low-grade Ga68-PSMA activity. CT-image **(A)**, PET image **(B)**, fused PET/CT image **(C)**, and the maximal intensity projection image **(D)**.

Low grade PSMA uptake has also been reported in the glandular tissue of the breasts in patients with gynecomastia (see Figure 6). This is based on PSMA expression on the epithelium of breast tissue (13).

Soft tissue hemangiomas may exhibit variable and even intense uptake. This is specifically problematic in the liver and spleen when trying to differentiate between hemangiomata and metastatic prostate carcinoma lesions. In such scenarios, the dynamic morphological features of hemangiomata may assist. During a triple phase contrast enhanced CT scan, hemangiomata will show typical slow, centripetal enhancement.

Benign neoplasms of the central and peripheral nervous system may demonstrate variable PSMA uptake. Meningiomas are usually seen in relation to the meninges, and typically are well-defined and of similar density as brain or slightly hyperdense and enhancing post contrast administration. Schwannomas may be found centrally or peripherally in relation to the spine, nerve roots, or peripheral nerves. They usually do not demonstrate local aggressiveness and are well circumscribed. Neurofibromas present with a typical subcutaneous soft tissue nodules (2, 4, 13).

### Malignant Neoplasms

**Figure 7:**
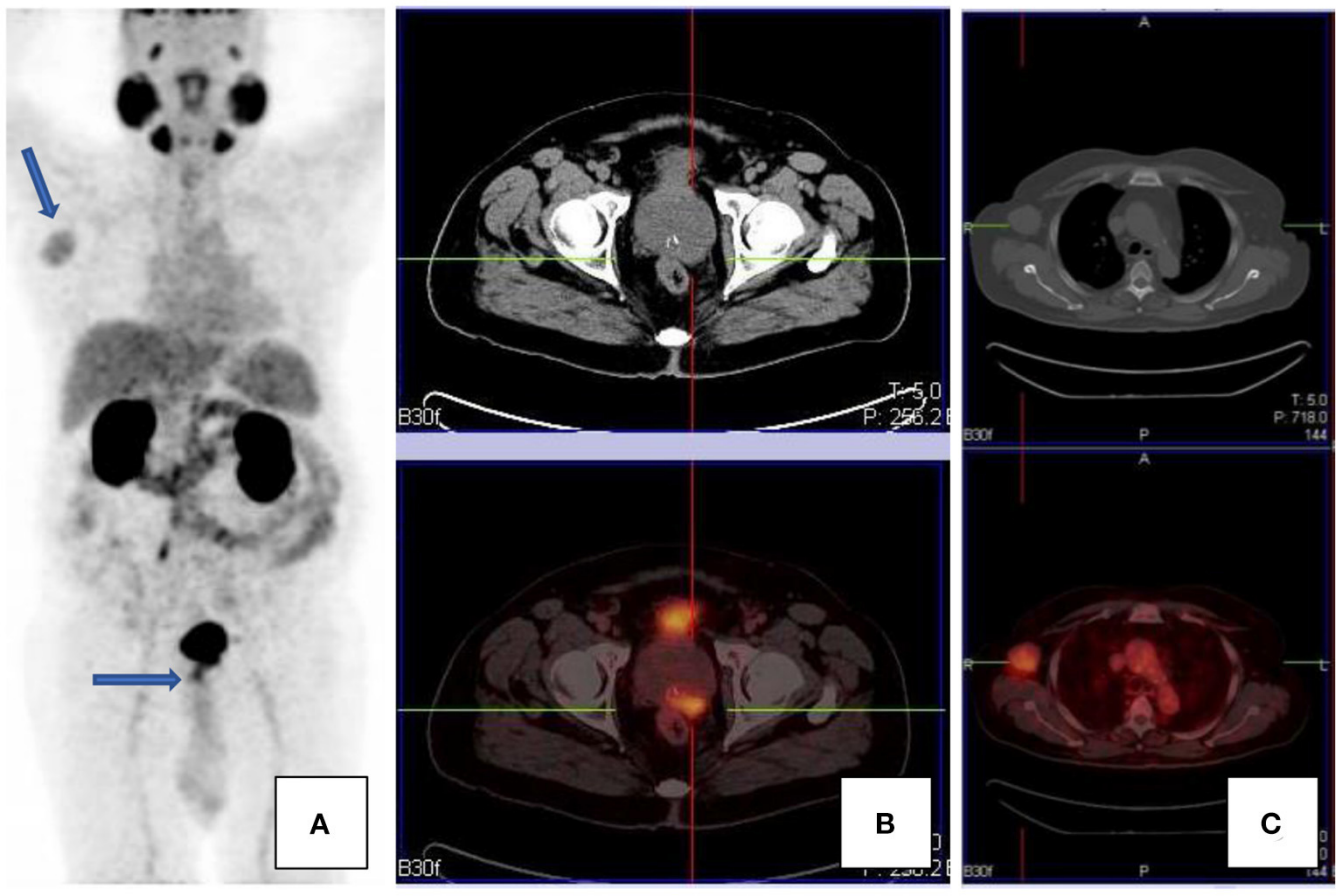
A patient with confirmed prostate carcinoma, with focal uptake in the prostate gland [transaxial CT and fused PET/CT images, **(B)**], as well as an avid right axillary nodal mass [transaxial CT and fused PET/CT images, **(C)**]. Maximal intensity projection images are shown in **(A)**. The axillary mass was an unlikely site of metastases, given the paucity of other metastases. The mass was excised and histology confirmed Hodgkin's lymphoma.

As was previously noted, PSMA expression is not specific to prostate cells. This includes tumors of the central nervous system, i.e., gliomas, thyroid cancer, breast, lung, lymphoma, neuroendocrine tumors, colorectal tumors, primary bone tumors, and many others (see Figure 7). Uptake in these tumors is related to PSMA expression and/or neovascularization. Renal cell carcinoma and transitional cell carcinoma may also demonstrate PSMA avidity; this is on the basis of intratumoral and peritumoral capillary endothelial cells which show intense immune reactivity since there is lack of PSMA expression. These entities contribute to false positive findings. One should always bear in mind the possibility of synchronous lesions, and imaging findings on scan should always be contextualized (2, 4, 13, 14).

## Artifacts

### Halo Artifacts

**Figure 8:**
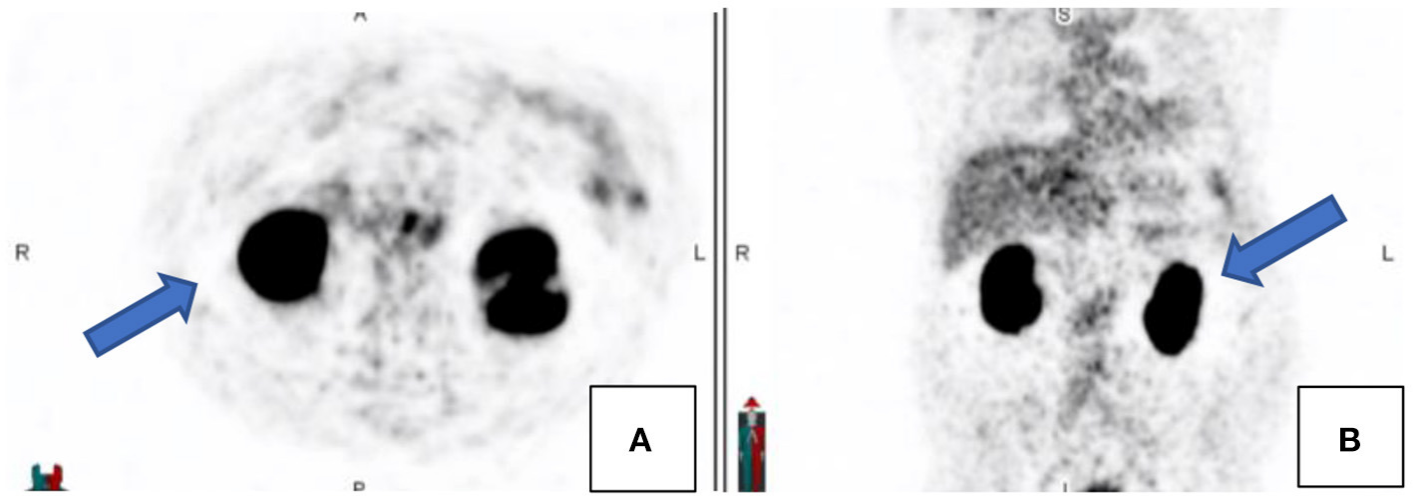
Transaxial **(A)** and coronal **(B)** PET images demonstrating the relative photopaenic areas surrounding the intense activity in the kidneys, the so-called “halo” effect, as indicated by the arrows.

Ga68-PSMA is excreted through the kidneys. This may result in high activity in the kidneys, urinary bladder, and associated structures which may cause a halo artifact and may impede visualization of lymph nodes peri-renal or peri-prostatic (see Figure 8).

This may be improved by various techniques. The administration of a diuretic agent like furosemide may assist in drainage or delayed imaging of the pelvic bed position after bladder voiding may also assist. When intravenous contrast is utilized, images in the delayed (urographic) phase may assist in these cases (6).

### Motion Artifacts

**Figure 9:**
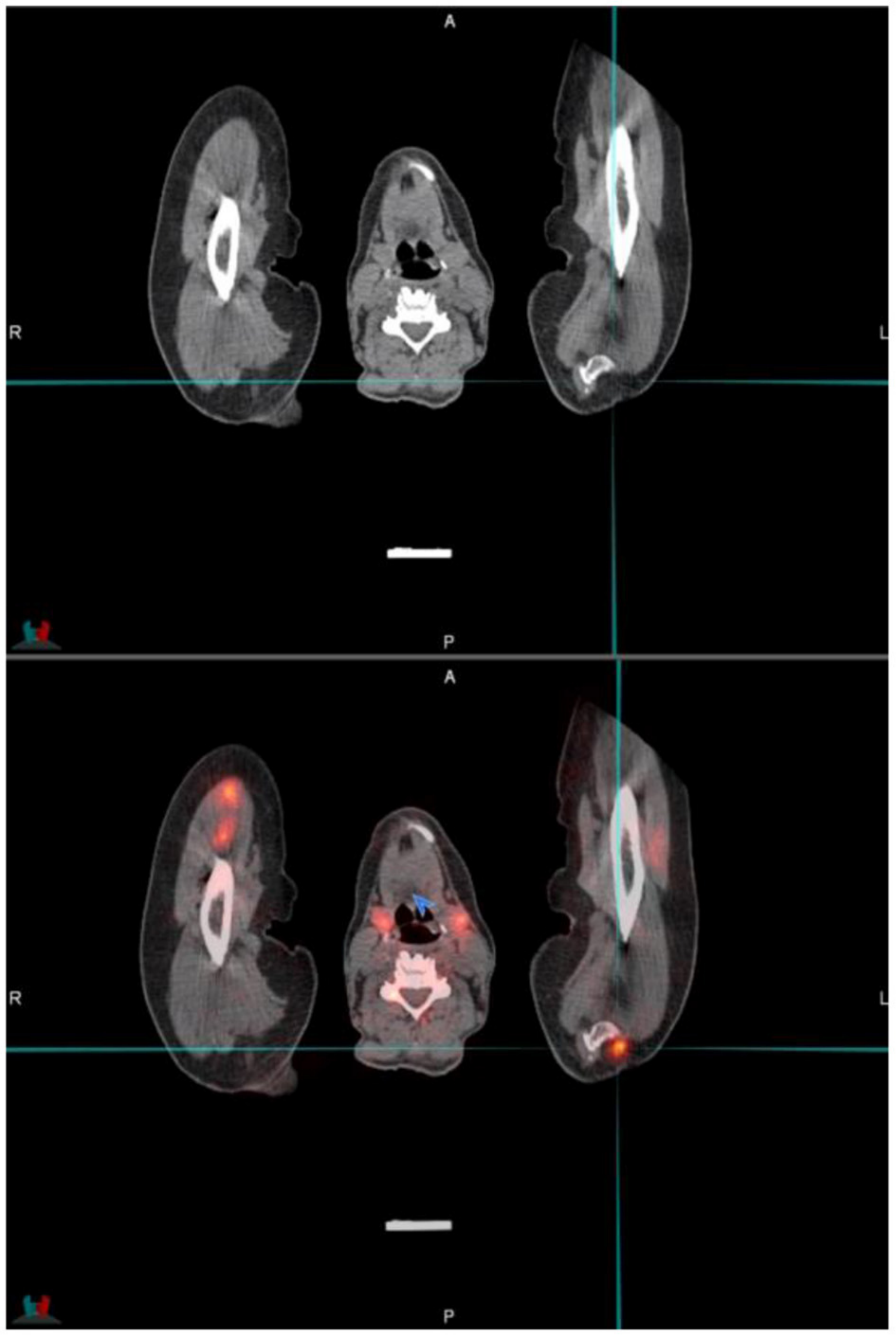
Transaxial CT and fused PET/CT images through the upper limbs and cervical area. The cross-hairs indicated a focus of activity that is misregistered due to patient movement of the arms—the activity should be located in the coracoid process of the left scapula that is in close proximity.

Patient motion and respiratory motion may lead to misregistration between anatomical detail and PSMA activity. This is more pronounced in the area in proximity to the diaphragm as well as patient extremities. Close inspection of the images is necessary, and usually the PET only images and maximal intensity projection images may provide valuable clues to possible misregistration (see Figure 9).

### PSMA Negative Prostate Cancer

**Figure 10:**
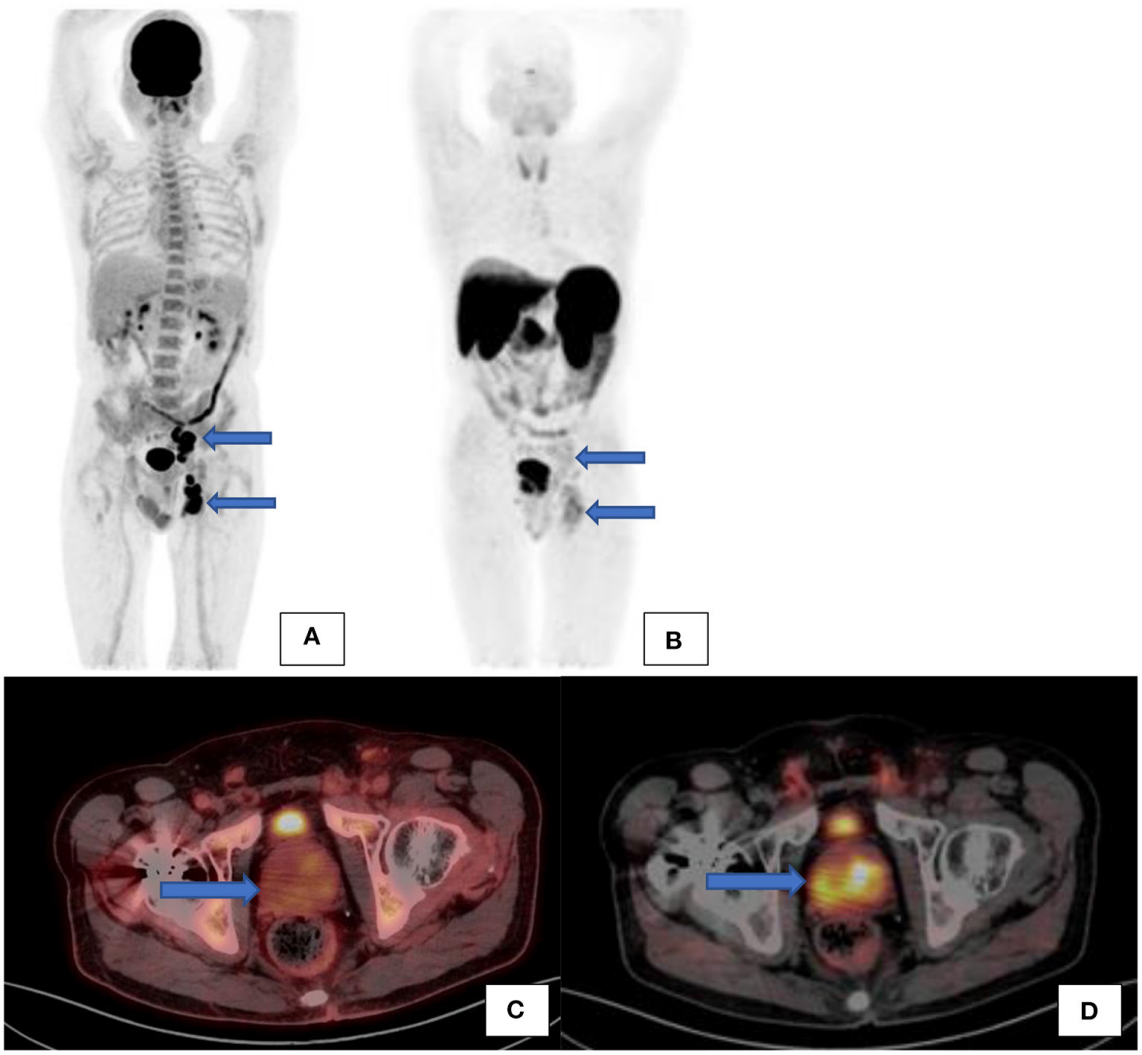
A patient with confirmed prostate carcinoma underwent a Ga68-PSMA scan (see the maximal intensity projection image **(B)** and transaxial fused PET/CT image **(D)** showing avid uptake in the prostate gland and low grade uptake in the left internal iliac and inguinal nodes. F18-FDG scan [**(A)**–maximal intensity projection images and **(C)**–transaxial fused PET/CT image] was also performed on the same patient showing low grade uptake in the prostate gland but avid uptake in the same left internal iliac and inguinal nodes.

Most prostate cancers are PSMA avid. It was reported that ~10% of prostate cancer may exhibit low grade PSMA expression and thus may not demonstrate avid uptake but only minimal or low-grade uptake. It is unlikely to encounter PSMA negative nodal metastases where the primary is PSMA avid, except in heavily chemotherapy pretreated patients (2) (see Figure 10). As prostate cancer is a heterogenous disease and primary tumor and metastases do not always show concordant PSMA expression, it has been shown that increasing PSMA percentage negativity of the primary tumor on immunohistochemistry is associated with an increasing rate of PSMA negative scans as well as PSMA negative metastases (15). In cases where there is evidence of biochemical recurrence, in view of rising PSA, with a negative PSMA scan then imaging with the following radiopharmaceuticals may be considered: 18F-fluciclovine (which is internalized into prostate cancer cells by amino acid transporters LAT1-4 and ASCT1/2 which are upregulated in prostate cancer) or 11C/18F-choline (which targets increased cell membrane lipid synthesis which is increased in cancer cells) (16).

### Flare Phenomenon

Following the initiation of androgen deprivation therapy (ADT) with a gonadotropin-releasing hormone (GnRH) antagonist, a heterogenous flare response may be noted, whereby lesions may show an increase in standardized uptake value of up to 73% after 2 weeks. Additional lesions may also be visible following the flare response, and the optimal period for lesion detection is 2–4 weeks after initiation of ADT (17).

### Confounding Factors: Iatrogenic Error

**Figure 11:**
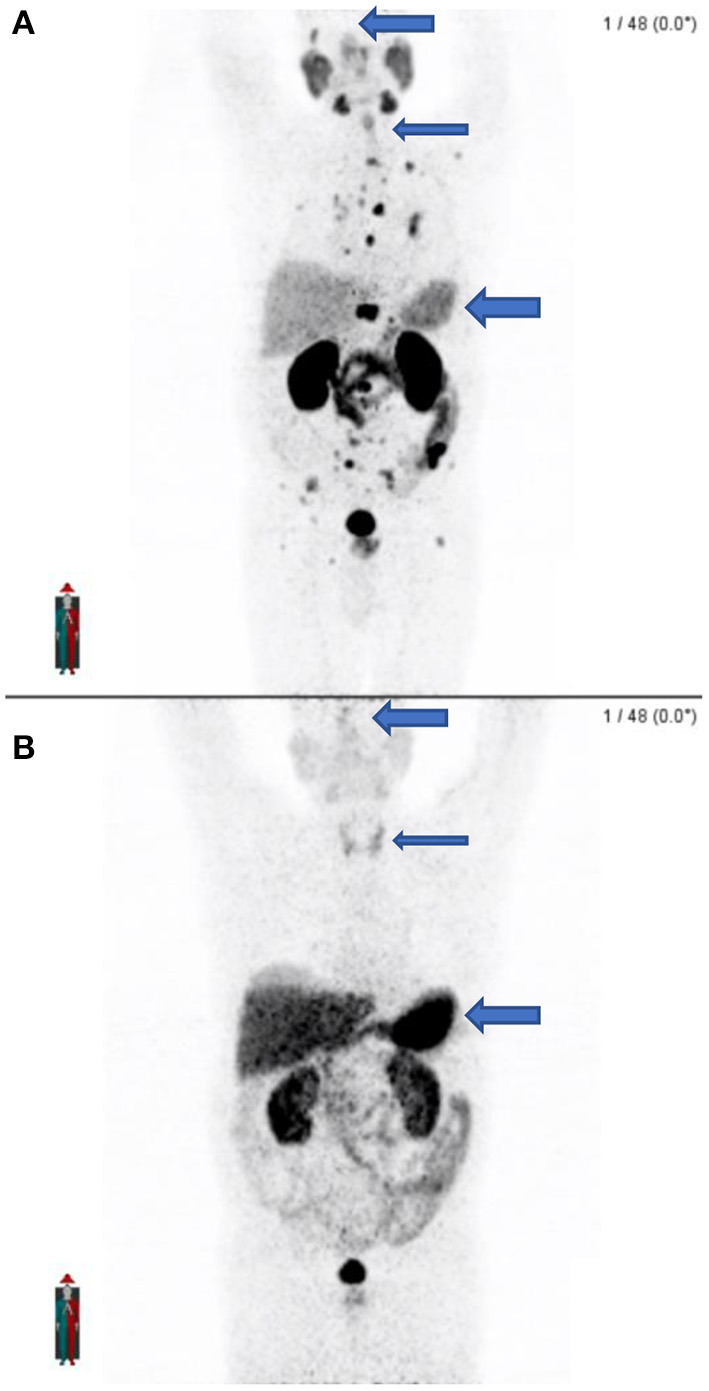
**(A)** shows the maximal intensity projection images of the patient after receiving the correct radiopharmaceutical (Ga68-PSMA) which shows widespread osteoblastic metastases. **(B)** Shows the initial maximal intensity projection image of the same patient 2 days earlier after receiving the incorrect radiopharmaceutical (Ga68-DOTATATE) with no apparent metastatic disease and different biodistribution. Note the lack of pituitary activity, minimal thyroid activity, and lesser hepatic and splenic activity on PSMA **(A)** as compared to the presence of pituitary activity, greater thyroid activity, and intense hepatic and splenic activity on DOTATATE **(B)**.

In busy departments where there is a high patient throughput and where a Ge68/Ga68 generator is used for inhouse preparation with cartridges or when patient doses are ordered, errors may occur. We noted unusual findings in two known prostate cancer patients that were scanned on the same day in our department. Upon close inspection of the MIP images, there was concern about what radiopharmaceutical was prepared. As both PSMA and DOTATATE labeled with Ga68 is prepared in the department, the suspicion was that DOTATATE instead of PSMA was prepared and injected into the patient. The images appeared to have an altered biodistribution with the pituitary noted, as well as the thyroid gland and intense uptake in the liver and spleen. This was unexpected biodistribution for a PSMA scan. Both patients were called to come back, and the scans were then repeated with PSMA within a few days. It was clear when comparing the PSMA scans to the initial scans that Ga68-DOTATATE was injected on the first visit (see Figure 11).

## Conclusion

The advent of Ga68-PSMA has revolutionized the imaging of prostate cancer since most prostate cancer does not show significant FDG avidity. Unfortunately, PSMA is not prostate specific and may accumulate in tissue other than prostate cancer due to PSMA expression of other cells, non-specific excretion, and neovascularization.

This contributes to various non-prostatic diseases which may demonstrate PSMA activity. From bone related conditions (fractures, osteomyelitis, multiple myeloma, Paget's disease, etc.) to inflammatory conditions (tuberculosis, sarcoidosis, diverticulosis, and others) to various benign and malignant conditions.

The reader should always contextualize findings, bear in mind that there may be synchronous lesions, and correlate findings against clinical history. Furthermore, a constant critical mind should be applied. A sound knowledge of the normal biodistribution of PSMA is essential as well as potential false negatives, pitfalls, and artifacts that may be encountered (see Table 1).

**Table 1:** Summary of artifacts and pitfalls associated with 68Ga-PSMA imaging.

**Artifacts** Halo artifact—intense renal activity causing photopaenia surrounding the kidneys Motion artifacts (respiratory and patient motion causing misregistration between PET and CT images)
**False negatives** PSMA negative prostate cancer (~10% of prostate carcinomas)
**Confounding factors** Iatrogenic errors (injection of wrong radiopharmaceutical)
**Flare phenomenon** (additional lesions visualized and increased SUV early after ADT)
**False positives** Infection/inflammation (pulmonary, prostatitis, post radiation etc.) Bone conditions (osteophytes, fractures, hemangiomas etc.) Benign neoplasms (related to vascular proliferation—e.g., thyroid, parathyroid, adrenal adenomas, etc.) Malignant neoplasms (tumors expressing PSMA—breast ca, lung ca, lymphoma, colorectal tumors, etc.)

This will ultimately lead to better reporting, fewer false negatives, and accurate imaging results. The reader should be mindful that other imaging modalities (MRI, triple phase contrast enhanced CT, and ultrasound) may assist in overall interpretation and potential problematic lesions.

## Author Contributions

Both authors listed have made a substantial, direct, and intellectual contribution to the work and approved it for publication.

## Conflict of Interest

The authors declare that the research was conducted in the absence of any commercial or financial relationships that could be construed as a potential conflict of interest.

## Publisher's Note

All claims expressed in this article are solely those of the authors and do not necessarily represent those of their affiliated organizations, or those of the publisher, the editors and the reviewers. Any product that may be evaluated in this article, or claim that may be made by its manufacturer, is not guaranteed or endorsed by the publisher.
